# Solid-state relaxation NMR dataset for a water-soluble β-(1→3, 1→6)-glucan from *Aureobasidium pullulans* and schizophyllan from *Schizophyllum commune*

**DOI:** 10.1016/j.dib.2019.104993

**Published:** 2019-12-12

**Authors:** Hiroyuki Kono, Nobuhiro Kondo, Takuya Isono, Makoto Ogata, Katsuki Hirabayashi

**Affiliations:** a Division of Applied Chemistry and Biochemistry, National Institute of Technology, Tomakomai College, Nishikioka 443, Tomakomai, Hokkaido 059 1275, Japan; b Itochu Sugar Co. Ltd, Tamatsuura 3, Hekinan, Aichi 447 8506, Japan; c Division of Biotechnology and Macromolecular Chemistry, Graduate School of Chemical Sciences and Engineering, Faculty of Engineering, Hokkaido University, Sapporo, Hokkaido 060 8628, Japan; d Department of Chemistry and Biochemistry, National Institute of Technology, Fukushima College, Nagao 30, Iwaki, Fukushima 970 8034, Japan

**Keywords:** β-(1→3, 1→6)-glucans, Solid-state NMR, *T*_1ρ_ relaxation, *T*_1_ relaxation, *Aureobasidium pullulans*, Schizophyllan, Molecular mobility, Triple helix

## Abstract

We report the solid-state nuclear magnetic resonance (NMR) relaxation dataset for a triple helix and a random structure of water-soluble *Aureobasidium pullulans* β-(1→3, 1→6)-d-glucan (APG) and those of schizophyllan from *Schizophyllum commune* (SPG), obtained by the Bruker BioSpin 500 MHz NMR spectrometer. These data include solid-state proton spin-lattice relaxation in the rotating frame (*T*_1ρH_) and ^13^C spin-lattice relaxation (*T*_1C_) of these two β-(1→3, 1→6)-glucans, which are related to the subject of article in *International Journal of Biological Macromolecules*, entitled “Characterization of the secondary structure and order–disorder transition of a β-(1→3, 1→6)-glucan from *Aureobasidium pullulans”* [1]. Data can help to investigate the structural characterization of the structural polysaccharides.

**Table:** Specifications Table

Subject	Polymers and Plastics
Specific subject area	Polysaccharides
Type of data	Analysed solid-state NMR data
How data were acquired	Bruker BioSpin AVIII 500 MHz NMR spectrometer equipped with Bruker BioSpin 4mm double-tuned MAS probe and TopSpin Ver 3.5 software for NMR data acquisition and processing.
Data format	Raw and analysed
Parameters for data collection	About 100 mg of each sample in ZrO_2_ rotor (4mm diameter) with Kel-F cap.
Description of data collection	All NMR experiments were performed at 298 K. Data were collected under magic angle spinning (MAS) frequency of 10 kHz.
Data source location	National Institute of Technology, Tomakomai College, Nishikioka 443, Tomakomai, Hokkaido 059 1275, Japan
Data accessibility	With the article
Related research article	Authors' name: Hiroyuki Kono*, Nobuhiro Kondo, Takuya Isono, Makoto Ogata, Katsuki Hirabayashi Title: Characterization of the secondary structure and order–disorder transition of a β-(1→3, 1→6)-glucan from *Aureobasidium pullulans* Journal: International Journal Biological Macromolecules https://doi.org/10.1016/j.ijbiomac.2019.11.018

**Table:** 

**Value of the Data** • The dataset is useful to characterize and understand the higher order structure of structural polysaccharides. • The dataset can be useful to researchers involved in the application of solid-state NMR in polymer chemistry and structural biology. • The dataset can be used as comparison in studies investigating the structural characterization of the other β-(1→3, 1→6)-glucans in the cell walls of cereals, bacteria, and fungi, with significantly differing physicochemical properties dependent on source. • To the best of our knowledge, this is the first published NMR relaxation dataset on *Aureobasidium pullulans* β-(1→3, 1→6)-glucan • The dataset would serve as a new analytical protocol for characterizing the formation of higher order structures of structural polysaccharides.

## 1 Data

The presented data include the *T*_1ρH_ and *T*_1C_ relaxation NMR data of a triple helix and a random structure of APG and those of SPG.^13^C NMR spectra of triple helix and a random structure of APG and those of SPG acquired by inserting 9 ^1^H spin-lock times that range from 0 to 15 ms during the *T*_1ρH_ experiments (Figs. S1–S4), and the ^13^C peaks were integrated for the following regions: C1 (110–96 ppm), C3 of the (1→3)-β-glucosyl main-chain (96–83 ppm), C6 (66–56 ppm), and other carbon resonances (83–66 ppm). The resulting integration values for each region as functions of ^1^H spin-lock time are summarized in Table 1, which were fitted to the following mono-exponential function:

(1) $$I_{t}=I_{0}\;\exp\left(-t/T_{1\text{ρ}H}\right)$$

where *I*_*t*_ is the measured integral value at ^1^H spin-lock time *t* and *I*_*0*_ is the initial intensity (*t* = 0) of the ^13^C magnetization. The *T*_1ρH_ values for the four ^13^C region of each sample could be determined by the fitting curves [1].

**Table 1:** Integration values for each region in the ^13^C spectra of APG and SPG samples as functions of ^1^H spin-lock time.

Sample	Spin-lock time/ms	C1	C3 (main-chain)	C2,3,4,5	C6
APG (triple helix)	0	0.163	0.112	0.640	0.085
	0.5	0.143	0.097	0.574	0.075
	1	0.128	0.089	0.512	0.068
	2	0.104	0.072	0.412	0.053
	3	0.080	0.058	0.330	0.042
	4	0.065	0.043	0.263	0.035
	8	0.023	0.016	0.111	0.012
	10	0.013	0.007	0.074	0.006
	15	0.002	−0.002	0.022	0.004
APG (random structure)	0	0.165	0.086	0.646	0.103
	0.5	0.150	0.074	0.585	0.091
	1	0.129	0.069	0.519	0.082
	2	0.105	0.051	0.412	0.068
	3	0.084	0.041	0.336	0.055
	4	0.068	0.027	0.271	0.040
	8	0.025	0.012	0.117	0.018
	10	0.021	0.007	0.074	0.009
	15	0.005	0.004	0.032	0.000
SPG (triple helix)	0	0.173	0.124	0.599	0.105
	0.5	0.153	0.112	0.544	0.094
	1	0.140	0.098	0.483	0.081
	2	0.110	0.080	0.389	0.068
	3	0.092	0.071	0.315	0.056
	4	0.074	0.055	0.257	0.044
	8	0.028	0.022	0.115	0.017
	10	0.018	0.013	0.073	0.010
	15	0.005	0.000	0.028	0.000
SPG (random structure)	0	0.169	0.120	0.600	0.111
	0.5	0.146	0.104	0.526	0.096
	1	0.124	0.087	0.456	0.084
	2	0.095	0.067	0.345	0.064
	3	0.070	0.047	0.265	0.049
	4	0.053	0.041	0.207	0.040
	8	0.013	0.010	0.070	0.010
	10	0.008	0.002	0.039	0.004
	15	0.001	−0.002	0.007	0.002

A series of ^13^C NMR spectra for a triple helix and a random structure of APG and those of SPG recorded with 10 relaxation delays that range from 0 to 60 s during the *T*_1C_ experiments (Figs. S5–S8). As described for the *T*_1ρH_ experiments, the four spectral regions were integrated (Table 2), and the resulting integration values for each region as functions of ^13^C relaxation delay were fitted to the following mono-exponential function:

(2) $$I_{t}=I_{0}\;\exp\left(-t/T_{1\text{C}}\right)$$

where *I*_*t*_ and *I*_*0*_ are defined as for Eq. (1). The *T*_1C_ values for the four ^13^C regions for each sample could be determined by the fitting curves [1].

**Table 2:** Integration values for each region in the ^13^C spectra of APG and SPG samples as functions of ^13^C relaxation delay.

Sample	Delay time/ms	C1	C3 (mainchain)	C2,3,4,5	C6
APG (triple helix)	0	0.163	0.112	0.640	0.085
	0.1	0.170	0.107	0.641	0.091
	0.5	0.178	0.112	0.637	0.085
	1	0.172	0.113	0.626	0.068
	2.5	0.162	0.108	0.592	0.044
	5	0.160	0.107	0.557	0.042
	7.5	0.155	0.103	0.522	0.032
	10	0.153	0.102	0.490	0.027
	30	0.113	0.081	0.327	0.012
	60	0.077	0.054	0.199	0.005
APG (random structure)	0	0.165	0.086	0.646	0.103
	0.1	0.164	0.078	0.651	0.099
	0.5	0.161	0.087	0.648	0.084
	1	0.164	0.084	0.645	0.071
	2.5	0.156	0.082	0.601	0.049
	5	0.151	0.086	0.564	0.039
	7.5	0.140	0.075	0.518	0.031
	10	0.136	0.073	0.475	0.026
	30	0.086	0.052	0.286	0.011
	60	0.053	0.026	0.166	0.002
SPG (triple helix)	0	0.173	0.124	0.599	0.105
	0.1	0.171	0.122	0.617	0.114
	0.5	0.171	0.126	0.604	0.090
	1	0.170	0.121	0.589	0.079
	2.5	0.170	0.120	0.557	0.050
	5	0.157	0.114	0.501	0.034
	7.5	0.151	0.113	0.464	0.024
	10	0.137	0.097	0.427	0.023
	30	0.094	0.076	0.250	0.006
	60	0.061	0.046	0.139	0.000
SPG (random structure)	0	0.169	0.120	0.600	0.111
	0.1	0.167	0.114	0.604	0.113
	0.5	0.172	0.121	0.595	0.095
	1	0.162	0.119	0.575	0.071
	2.5	0.156	0.112	0.533	0.038
	5	0.143	0.107	0.471	0.030
	7.5	0.125	0.091	0.405	0.015
	10	0.111	0.083	0.361	0.010
	30	0.060	0.046	0.158	0.000
	60	0.023	0.018	0.059	0.000

## 2 Experimental design, materials, and methods

APG was kindly provided from Itochu Sugar Co. (Japan), which was prepared according to a previously reported method [[2], [3], [4]]. SPG was purchased from InvivoGen (USA). Triple helical and single random coil structures of APG were prepared by dissolving 250 mg of APG in 50 mL of deionized water and DMSO, respectively, at 298 K for 3 d followed by lyophilization. In a method similar to that used to prepare the triple helical and single random coil structures of APG, lyophilization of SPG dissolved in water or DMSO for 3 d provided the triple helical and single random coil structures of SPG, respectively [1].

Solid-state *T*_1ρH_ and *T*_1C_ experiments were performed at 298 K using a Bruker AVIII500 spectrometer (Bruker BioSpin GmbH, Germany) equipped with a 4 mm dual-tuned MAS probe according to methods previously reported [5,6]. To determine *T*_1ρH_ values, Cross-polarization (CP)/MAS ^13^C NMR spectra were recorded by inserting ^1^H spin-lock times of 0.5, 1, 2, 3, 4, 8, 10, and 15 ms prior to CP, and MAS frequency, contact time, acquisition time, and repetition time were set to 10 kHz, 2 ms, 15 ms, and 4 s, respectively. The *T*_1ρH_ values for the specific ^13^C resonance regions were integrated to obtain the *T*_1ρH_ curves, which were fitted to Eq. (1). The *T*_1C_ experiments were performed using the Torchia method [7]. The spectra were recorded at relaxation delays of 0.1, 0.5, 1, 2.5, 5, 7.5, 10, 30, and 60 s, and the specific ^13^C resonance regions were integrated to obtain *T*_1C_ curves, which were fitted to Eq. (2). The chemical shifts were calibrated by assigning the value of 176.03 ppm to the carbonyl carbon of the external standard d-glycine.

## Funding

This work was, in part, supported by the Japan Society for Promotion of Science (JSPS) [grant number JP16K05802] (H.K.).

## Conflict of Interest

The authors declare that they have no known competing financial interests or personal relationships that could have appeared to influence the work reported in this paper.
