# Supplementary material for: Solid-state relaxation NMR dataset for a water-soluble β-(1→3, 1→6)-glucan from Aureobasidium pullulans and schizophyllan from Schizophyllum commune
Source: Data Brief. 2019 Dec 12;28:104993. doi: 10.1016/j.dib.2019.104993 (PMC6933183; doi:10.1016/j.dib.2019.104993)
Supplement: Multimedia component 1 [file mmc1.pdf]

Appendix A. Supplementary material

Solid-state relaxation NMR dataset for a water-soluble β-(1→3, 1→6)-glucan from *Aureobasidium pullulans* and schizophyllan from *Schizophyllum commune*

Authors

Hiroyuki Kono[1]*, Nobuhiro Kondo[2], Takuya Isono[3], Makoto Ogata[4], Katsuki Hirabayashi[2]

Affiliations

1. Division of Applied Chemistry and Biochemistry, National Institute of Technology, Tomakomai College, Nishikioka 443, Tomakomai, Hokkaido 059 1275, Japan

2. Itochu Sugar Co. Ltd, Tamatsuura 3, Hekinan, Aichi 447 8506, Japan

3. Division of Biotechnology and Macromolecular Chemistry, Graduate School of Chemical Sciences and Engineering, Faculty of Engineering, Hokkaido University, Sapporo, Hokkaido 060 8628, Japan

4. Department of Chemistry and Biochemistry, National Institute of Technology, Fukushima College, Nagao 30, Iwaki, Fukushima 970 8034, Japan

Corresponding author

Hiroyuki Kono (kono@tomakomai-ct.ac.jp)

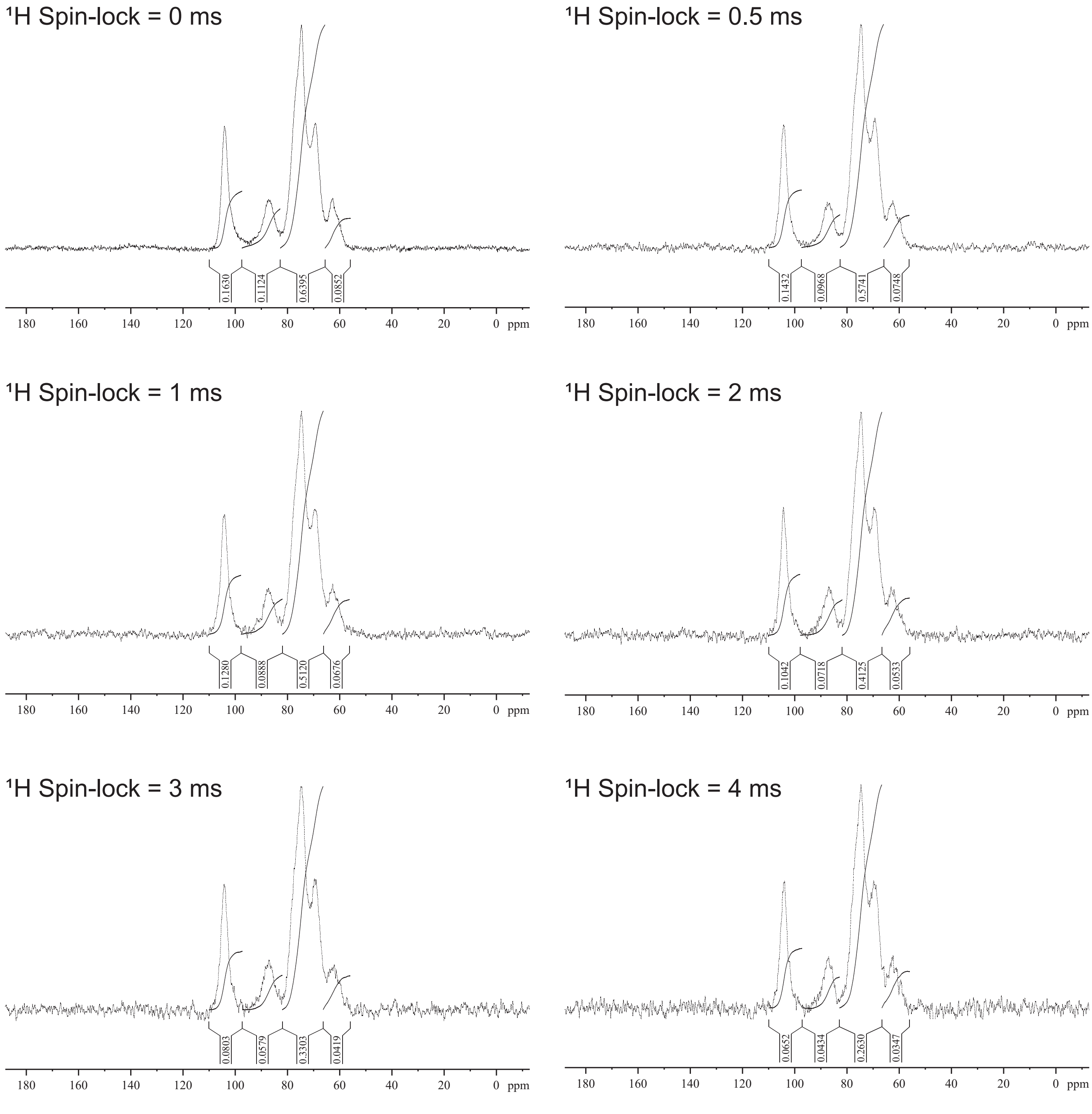

Fig. S1.
^{13}C spectra of APG (triple helix) recorded by inserting 9 ^{1}H spin-lock times during the $T_{1\rho H}$ experment.

^{1}H Spin-lock = 8 ms

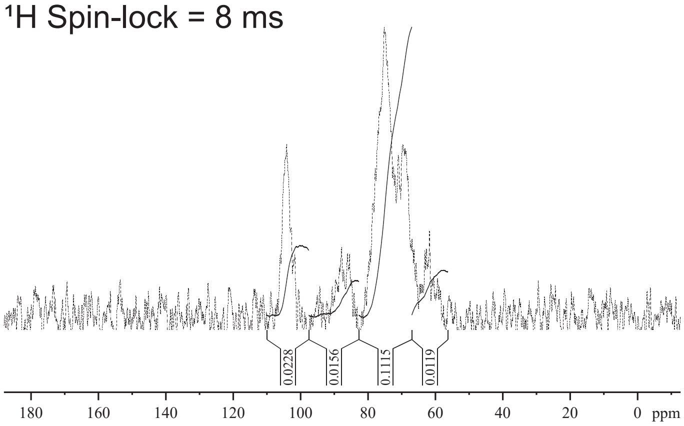

^{1}H Spin-lock = 10 ms

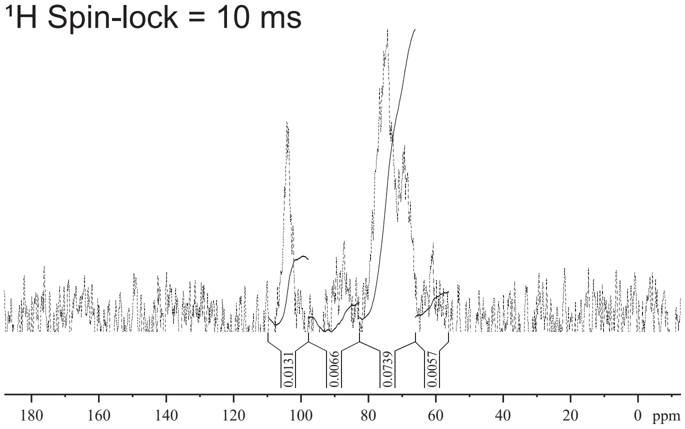

^{1}H Spin-lock = 15 ms

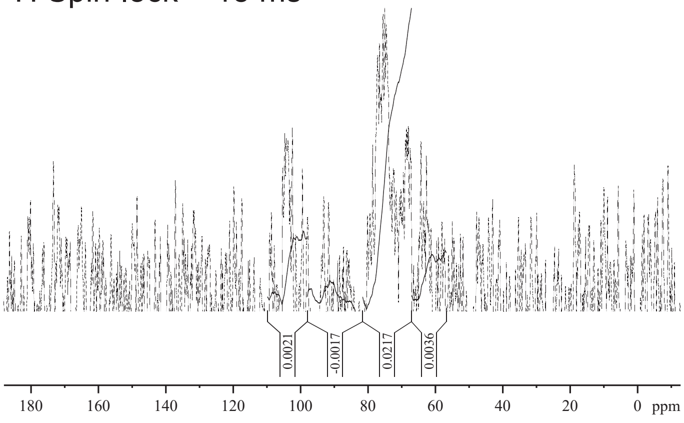

Fig. S1.
(*continued*)

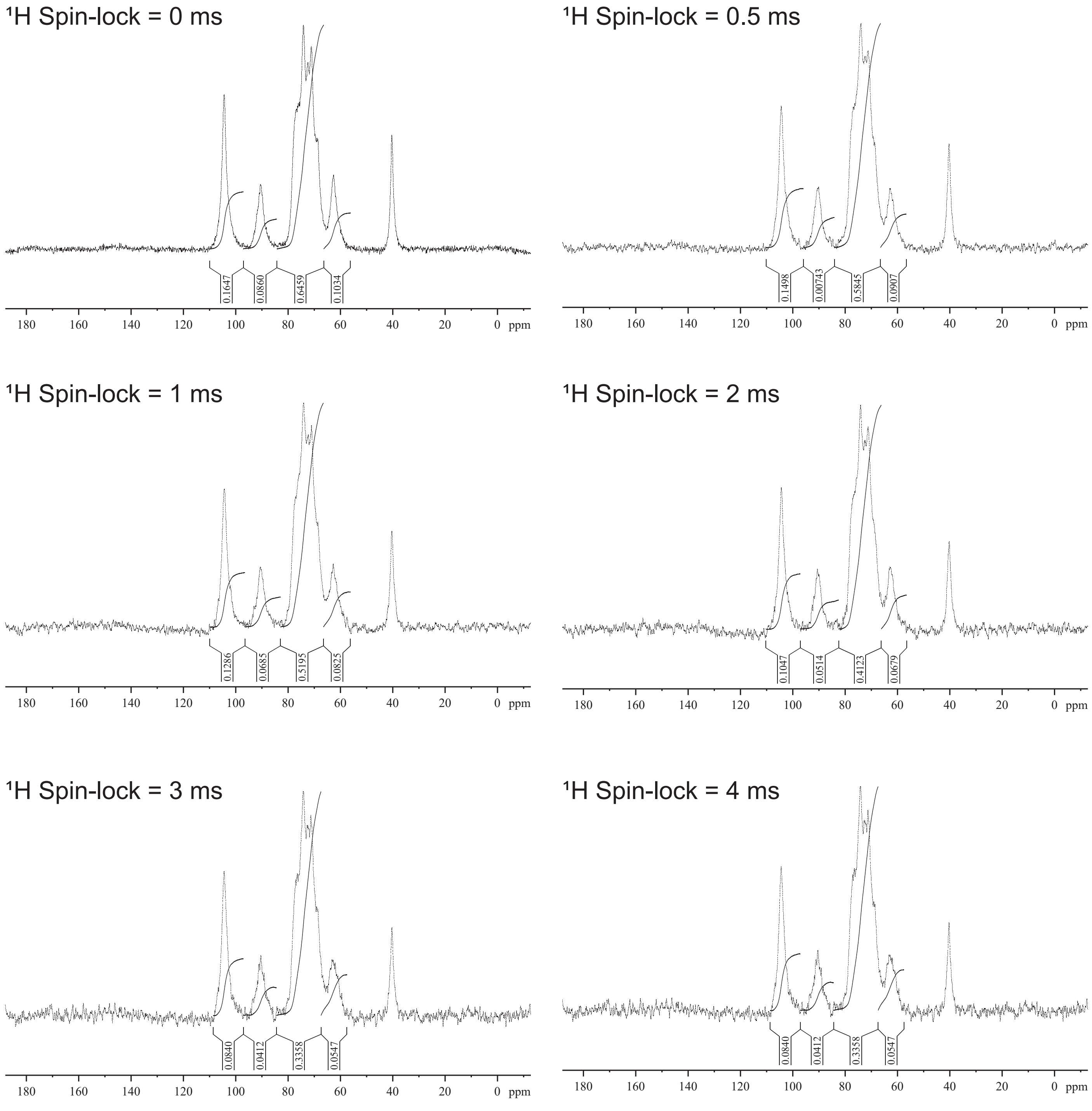

Fig. S2.
^{13}C spectra of APG (random structure) recorded by inserting 9 ^{1}H spin-lock times during the $T_{1\rho H}$ experment.

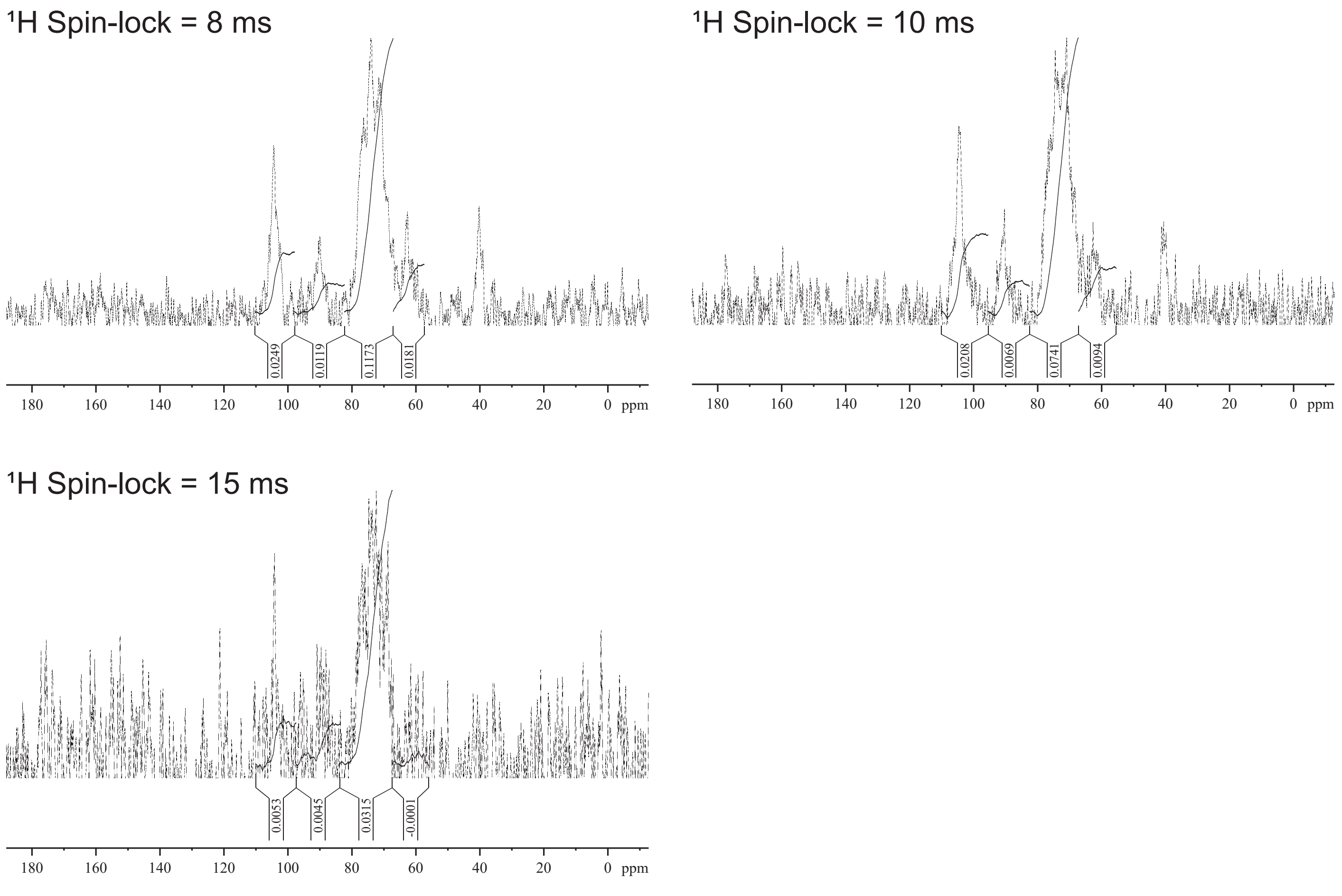

Fig. S2.
(*continued*)

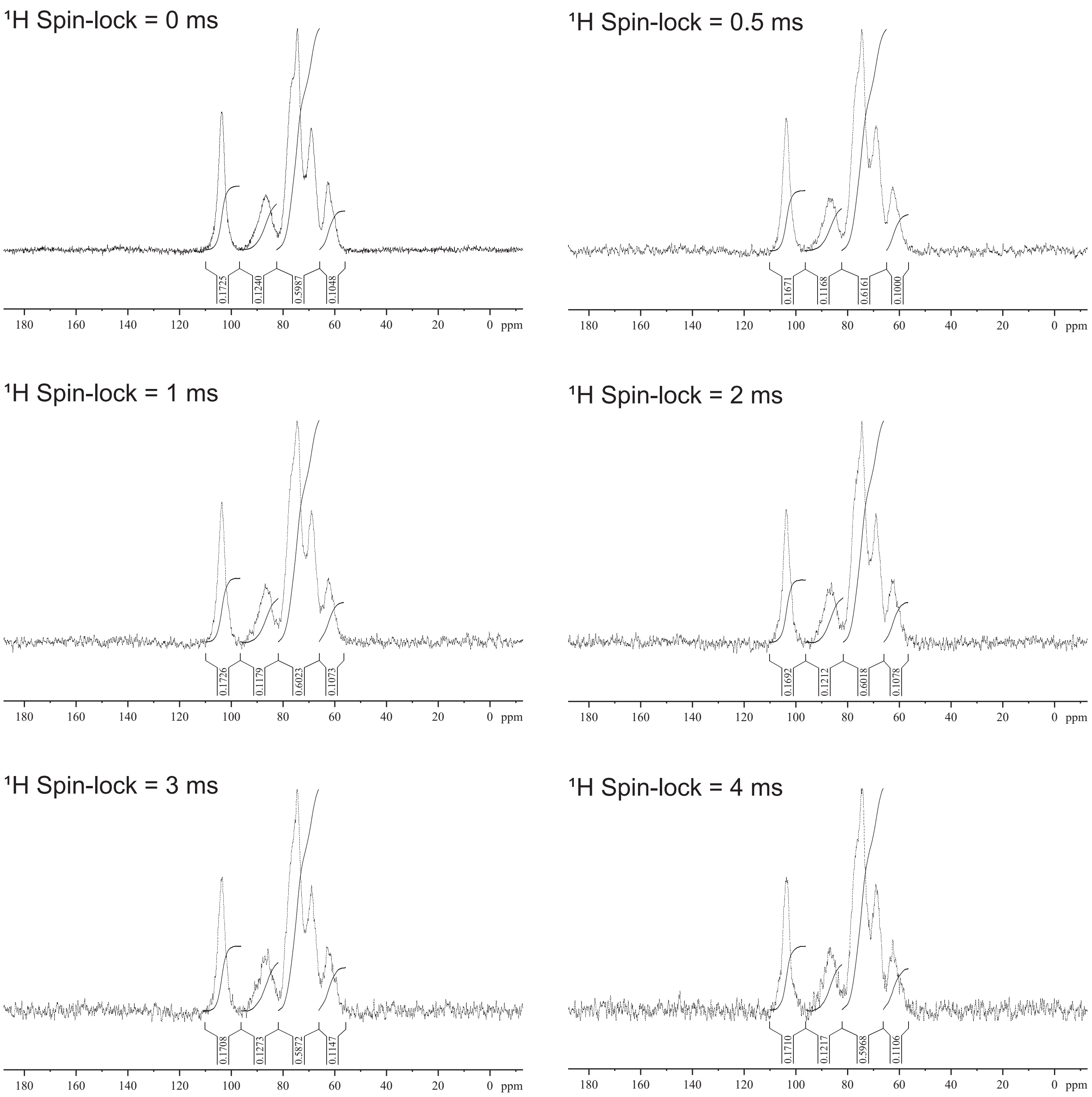

Fig. S3.
^{13}C spectra of SPG (triple helix) recorded by inserting 9 ^{1}H spin-lock times during the $T_{1\rho H}$ experment.

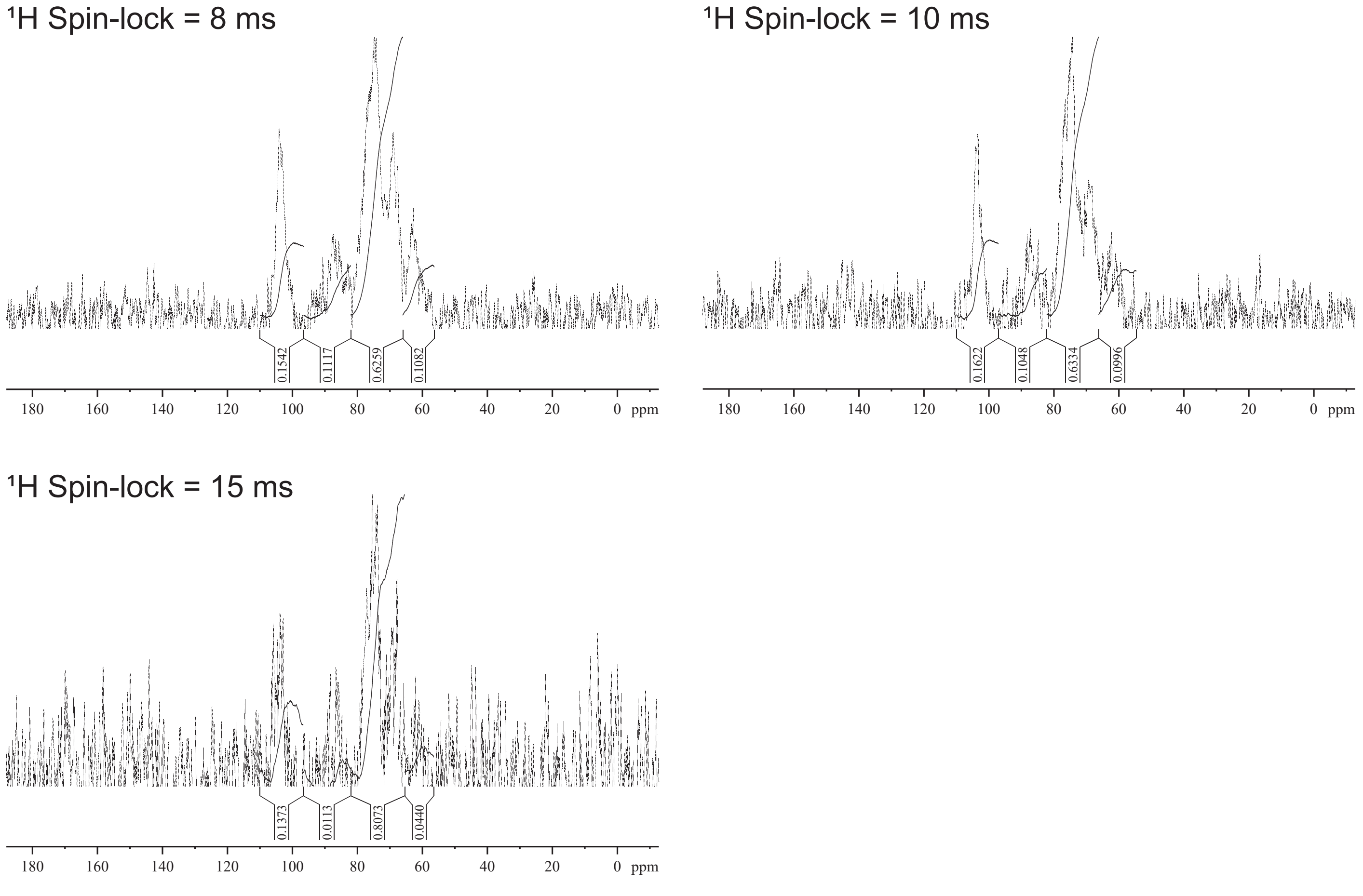

Fig. S3.
(*continued*)

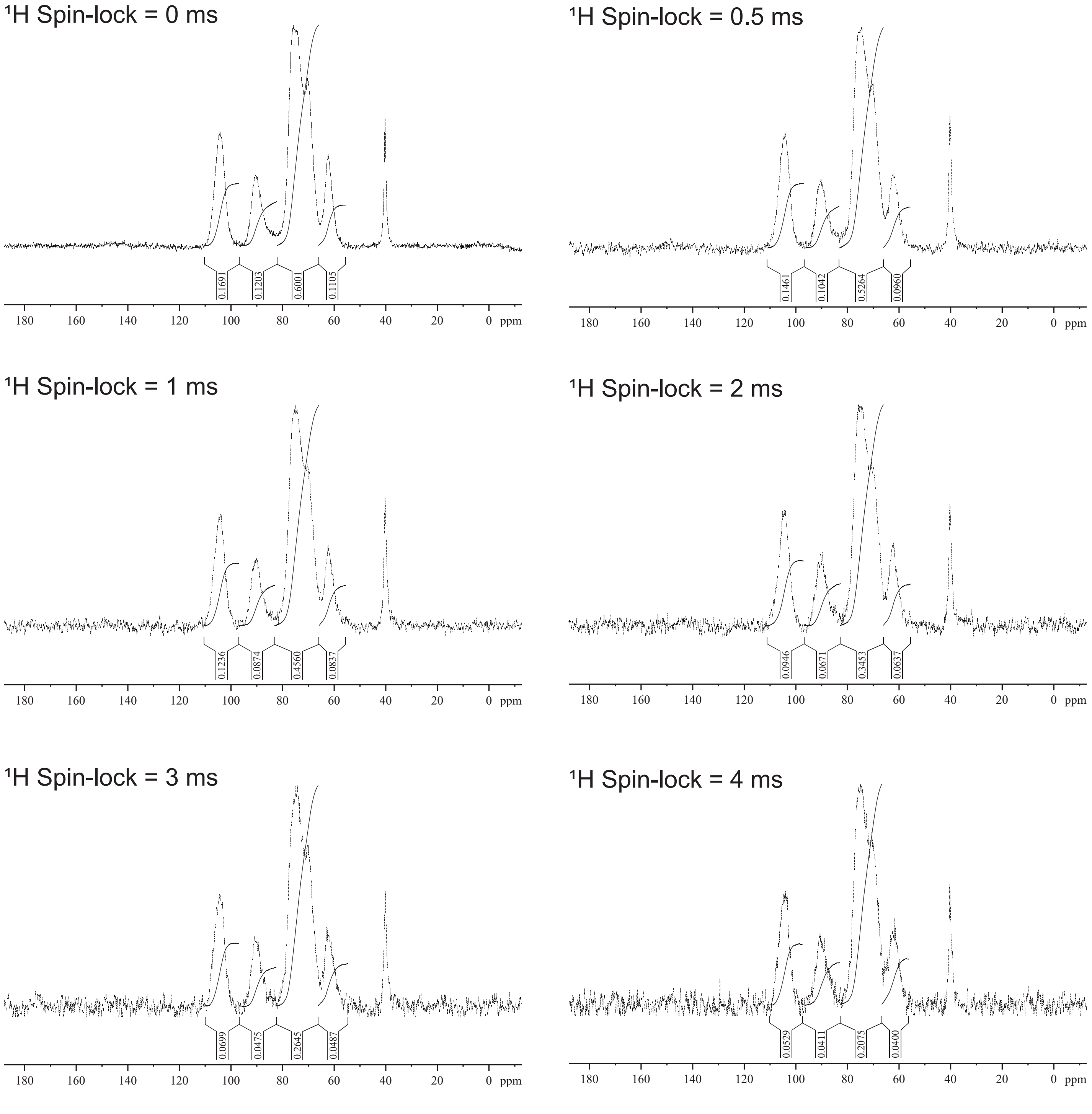

Fig. S4.

^{13}C spectra of SPG (random structure) recorded by inserting 9 ^{1}H spin-lock times during the $T_{1\rho H}$ experment.

^{1}H Spin-lock = 8 ms

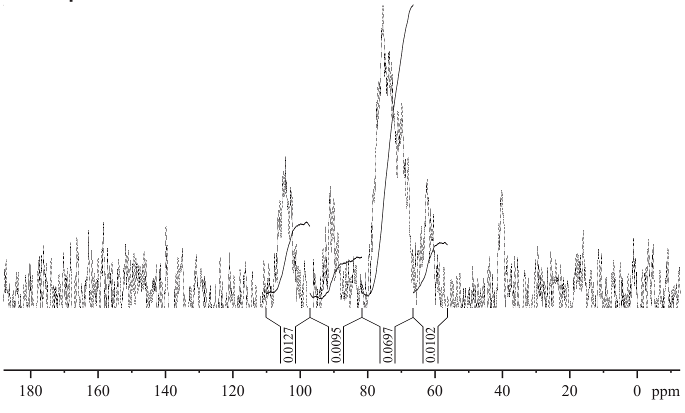

^{1}H Spin-lock = 10 ms

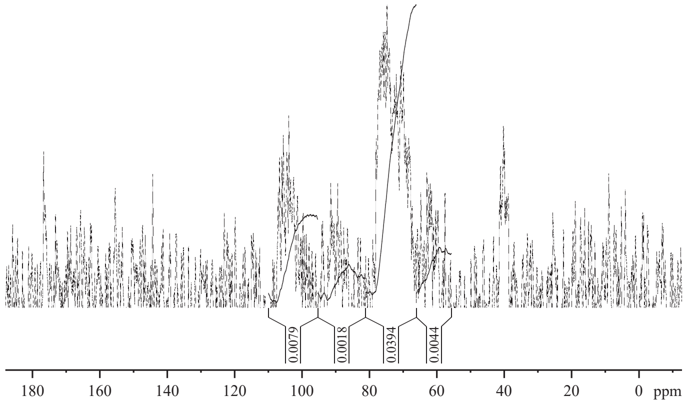

^{1}H Spin-lock = 15 ms

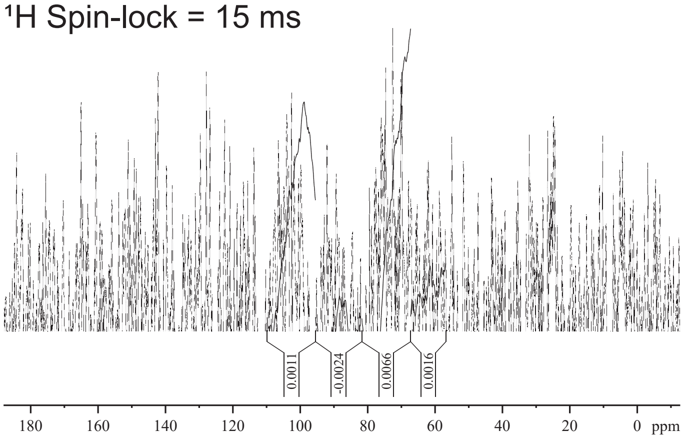

Fig. S4.
(*continued*)

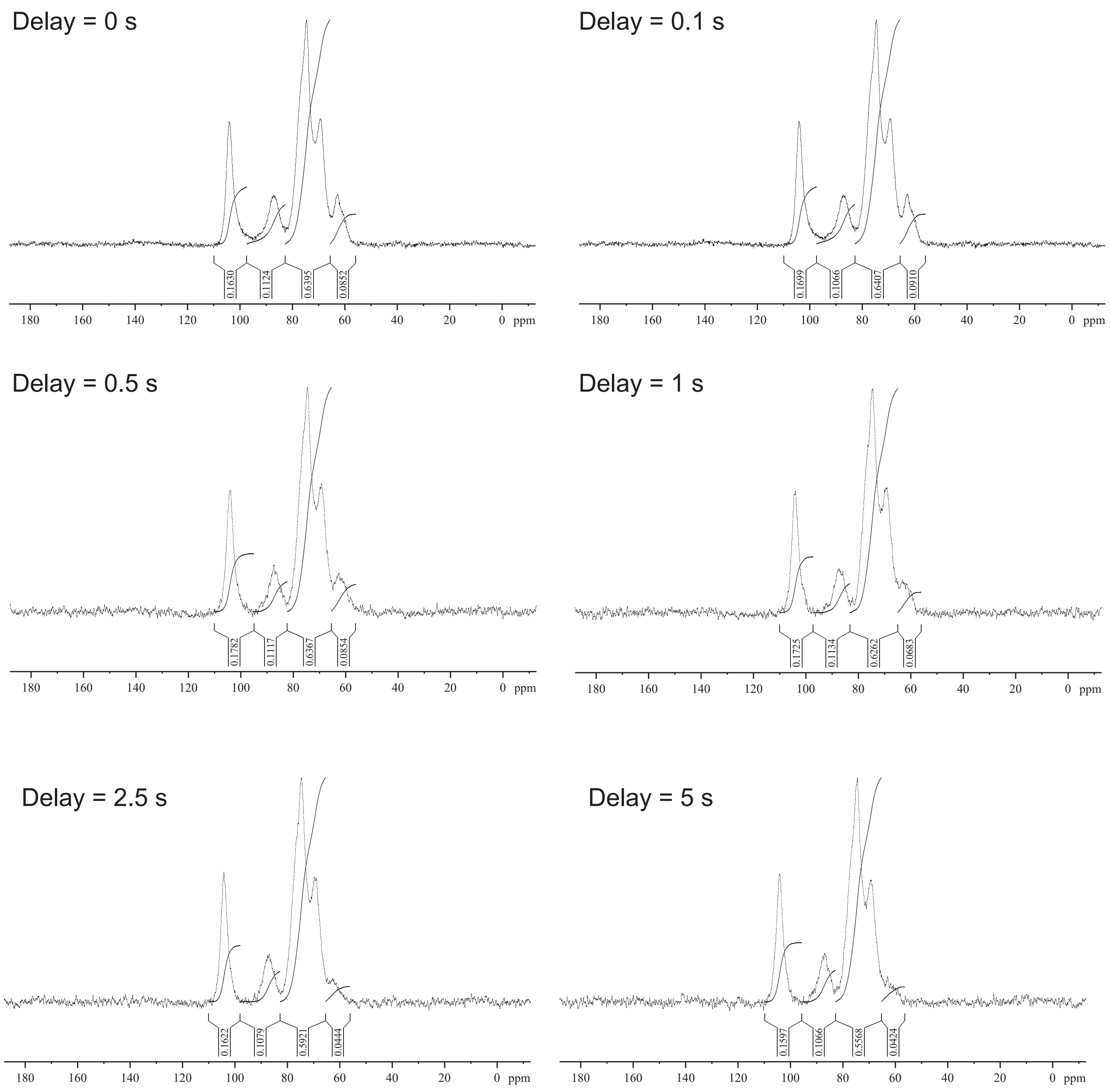

Fig. S5.
^{13}C spectra of APG (triple helix) recorded with 10 relaxation delays during the T_{1C} experment.

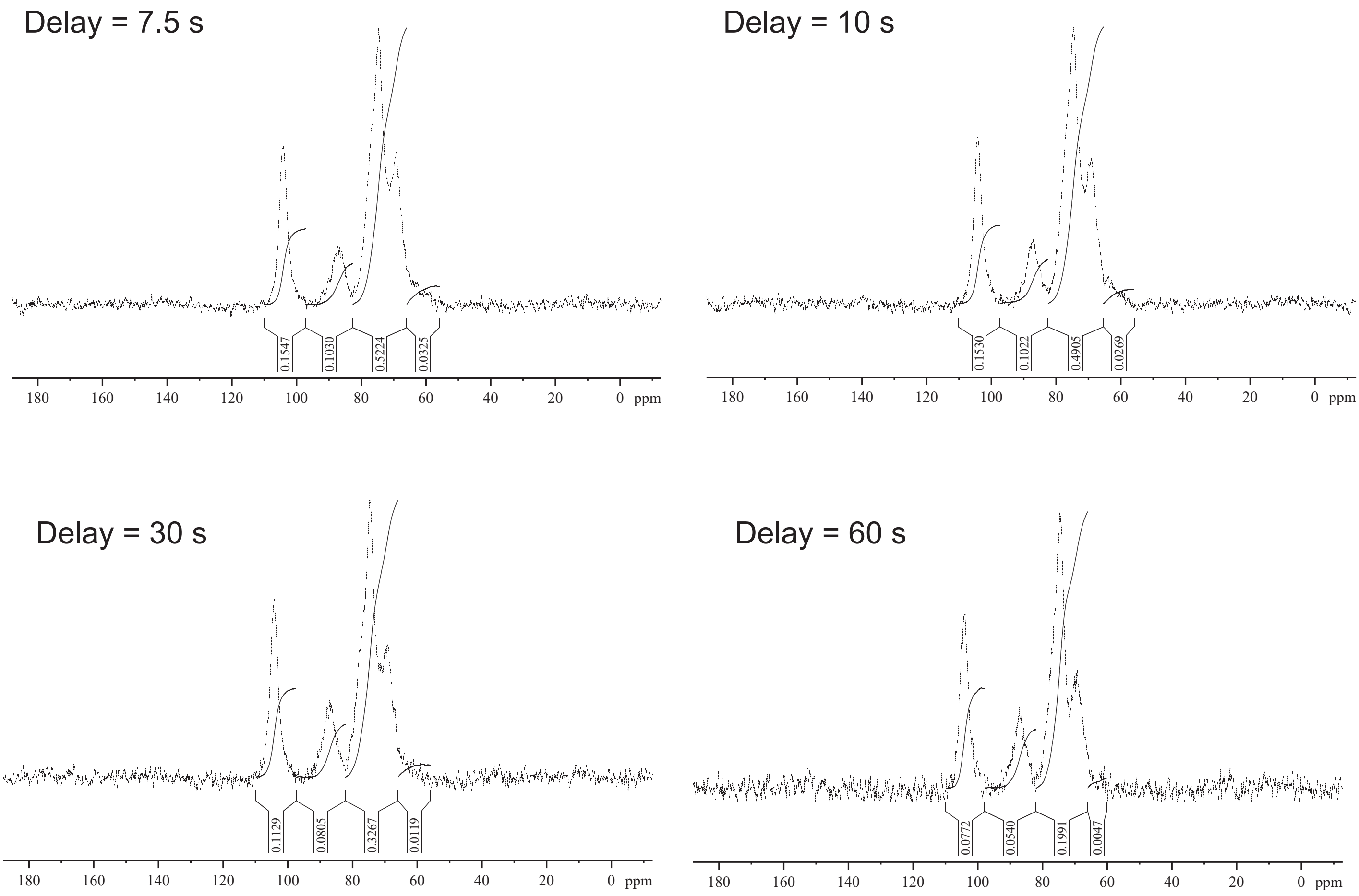

Fig. S5.
(*continued*)

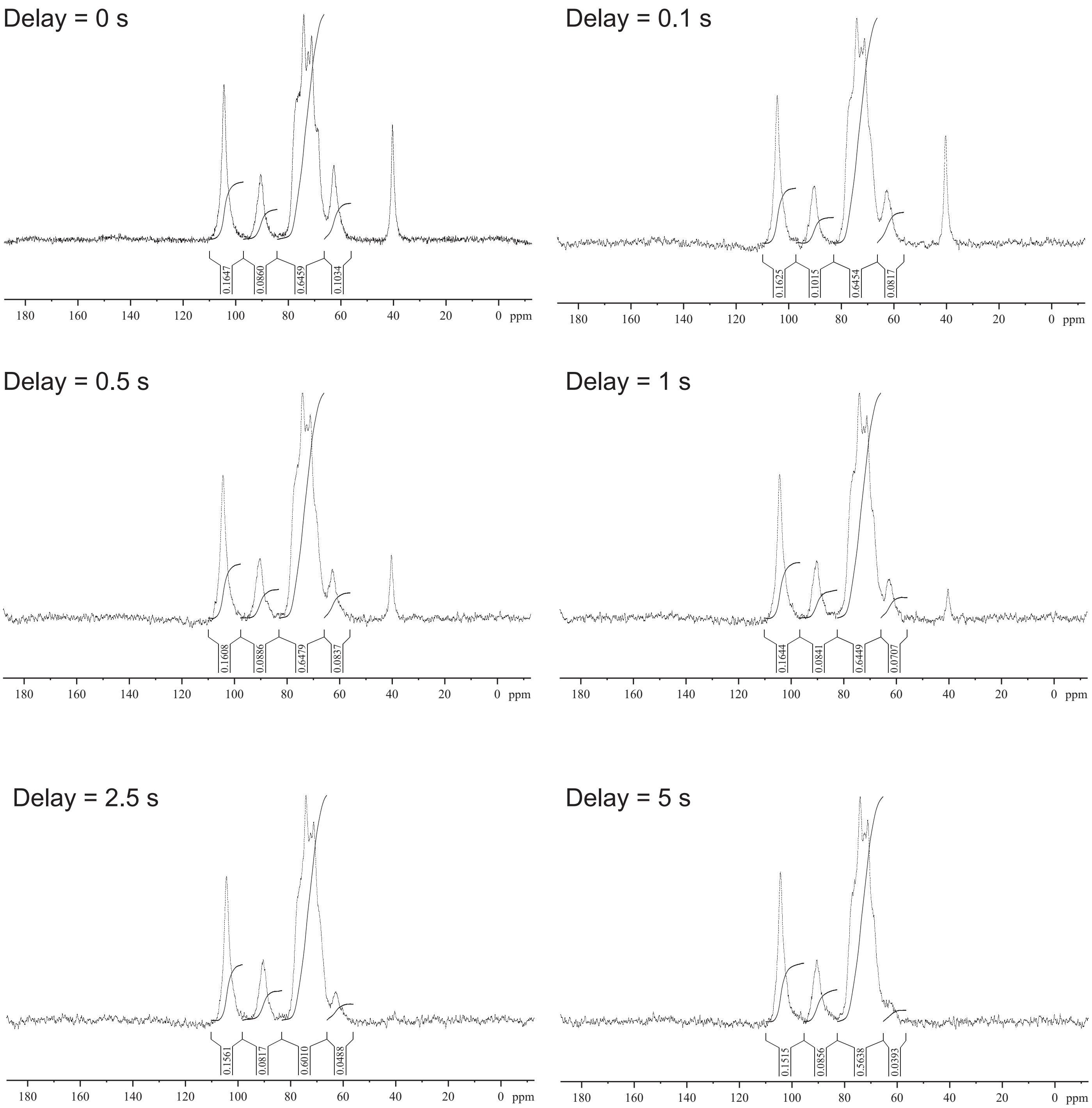

Fig. S6.
^{13}C spectra of APG (random structure) recorded with 10 relaxation delays during the T_{1C} experment.

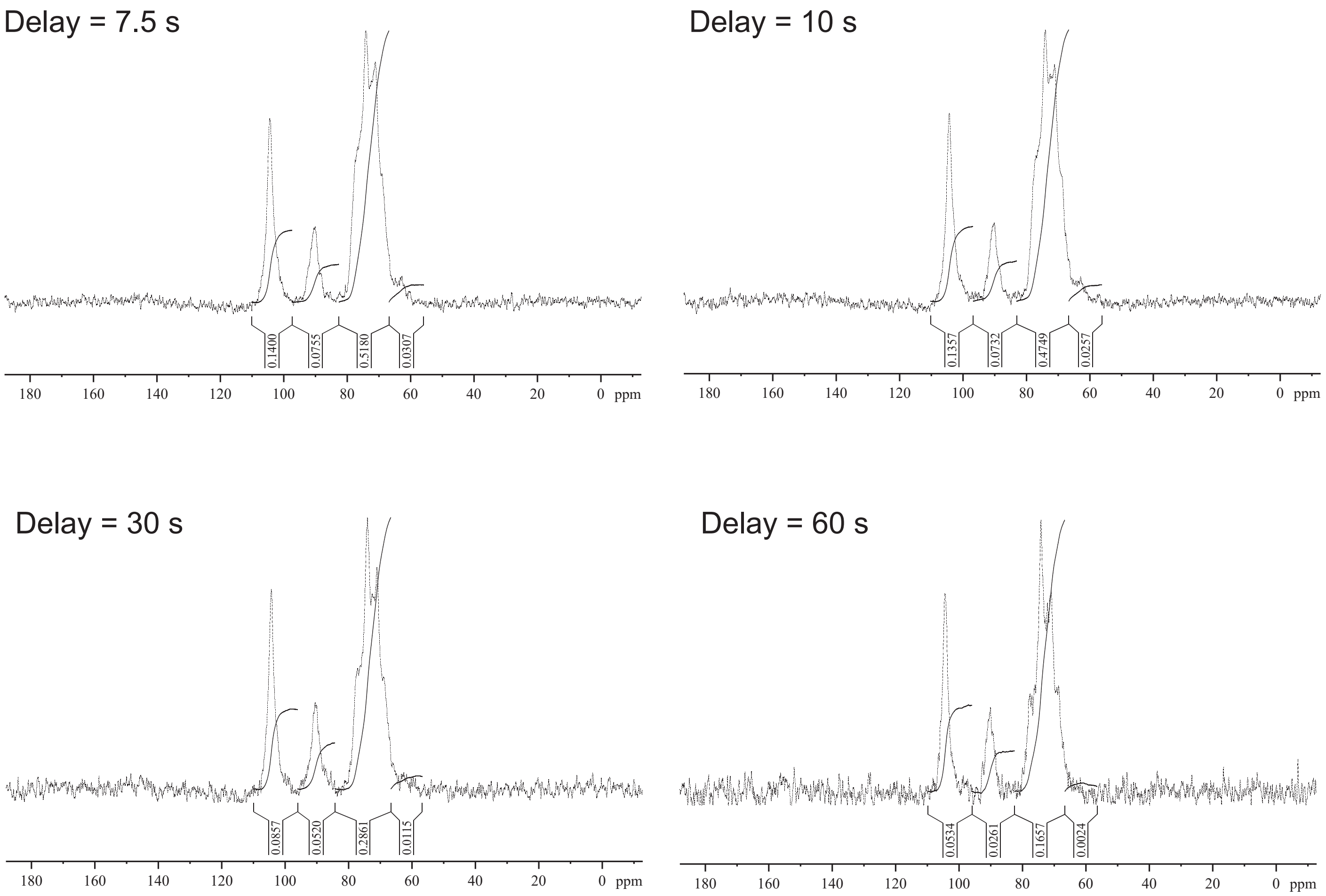

Fig. S6.
(*continued*)

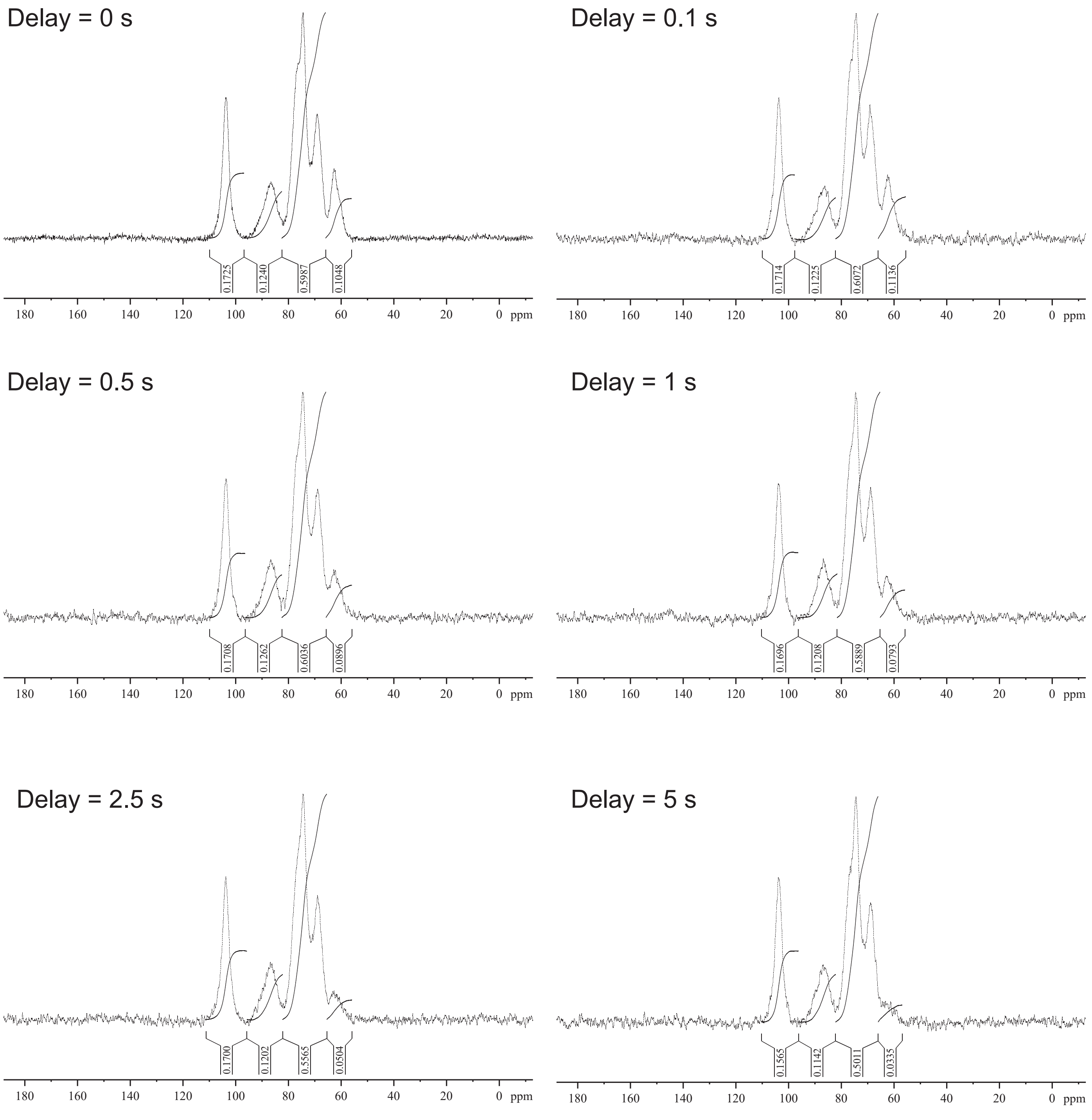

Fig. S7.

^{13}C spectra of SPG (triple helix) recorded with 10 relaxation delays during the T_{1C} experment.

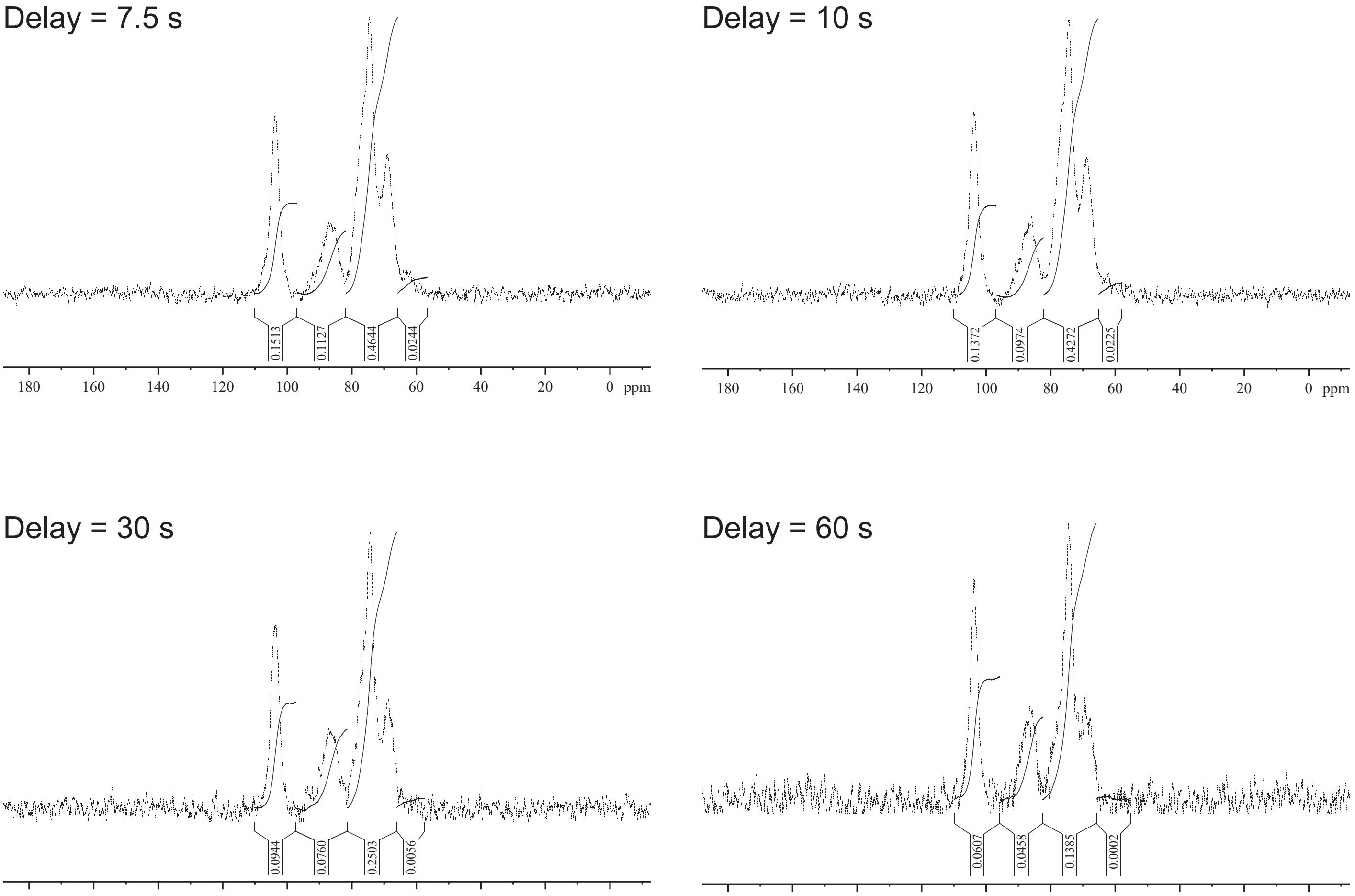

Fig. S7.
(*continued*)

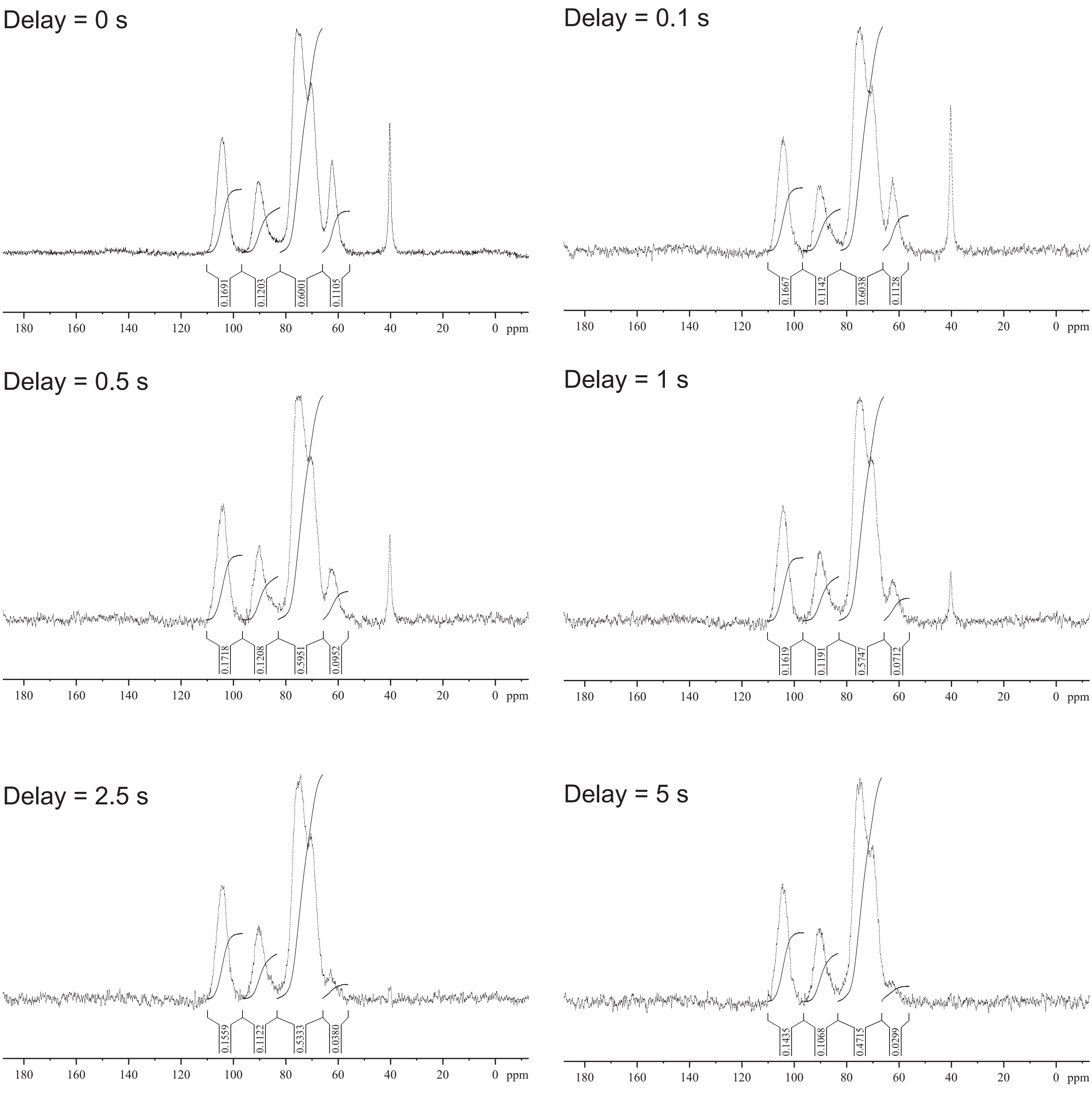

Fig. S8.
^{13}C spectra of SPG (random structure) recorded with 10 relaxation delays during the T_{1C} experment.

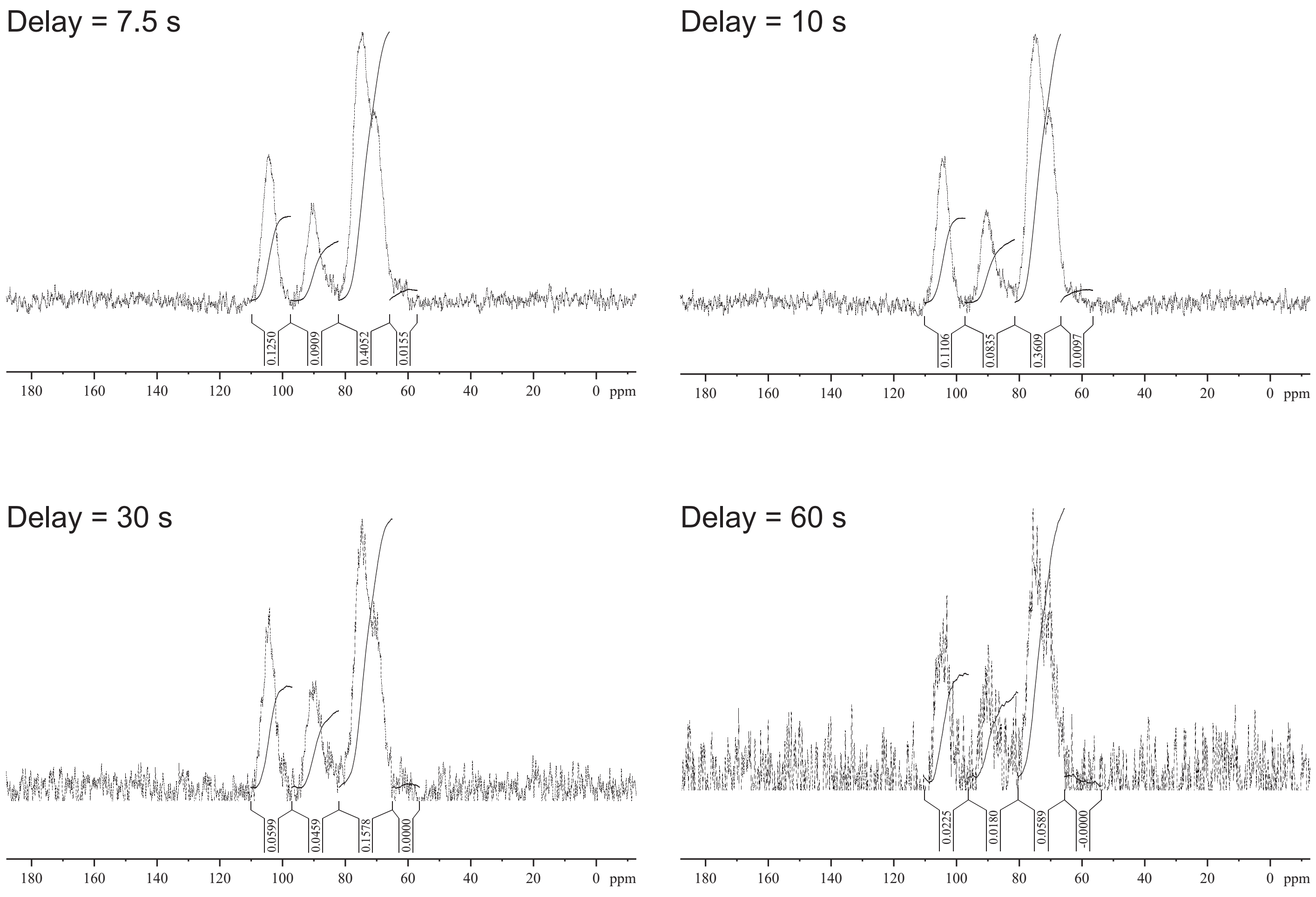

Fig. S8.
(*continued*)
